# A Study of Sputum Conversion in New Smear Positive Pulmonary Tuberculosis Cases at the Monthly Intervals of 1^st^, 2^nd^ & 3^rd^ Month Under Directly Observed Treatment, Short Course (Dots) Regimen

**DOI:** 10.4103/0970-2113.44122

**Published:** 2008

**Authors:** S Bawri, S Ali, C Phukan, B Tayal, P Baruwa

**Affiliations:** 1 Post Graduate, Deptt. of Medicine, Gauhati Medical College, Guwahati, Assam; 2 Prof., Deptt. of Medicine, Gauhati Medical College, Guwahati, Assam; 3 Asstt. Prof., Gauhati Medical College, Guwahati, Assam; 4 Junior Resident, Deptt. of Medicine, Gauhati Medical College, Guwahati, Assam; 5 HOD & Professor, Deptt. of Chest & TB, Gauhati Medical College, Guwahati, Assam

**Keywords:** Sputum Conversion, 1st, 2nd & 3rd Month, DOTS & RNTCP

## Abstract

**Aims and Objectives:**

To determine sputum conversion rate at monthly intervals of 1st, 2nd and 3rd month in new smear positive cases (cat-1) under treatment under RNTCP.

**Material and Methods:**

The study was conducted at DOTS Center, Gauhati Medical College and Hospital; Guwahati between July 2005 to June 2006.The study is a prospective study and consists of 100 cases of new smear positive pulmonary tuberculosis cases (category 1) irrespective of age and sex.

**Results and Observations:**

The age & sex distribution of 100 patients showed that majority of the patients (74%) belonged to 2nd, 3rd and 4th decades & 75% were males and 25% were female with male to female ratio 3:1 respectively. The chest x-ray of 100 Smear Positive patients shows that only 60 (60%) patients had x-ray evidence of pulmonary Koch. In the present study, sputum conversion i.e. from smear positive to smear negative at the end of the 1st month is 71%, at the end of 2nd month is 84% and at the end of 3rd month is 92%.

**Summary & Conclusion:**

In conclusion, the overall sputum conversion rate under Directly Observed Treatment, Short Course (DOTS) chemotherapy in 100 sputum smear positive Pulmonary Tuberculosis in DOTS centre, Gauhati Medical College & Hospital was 92%.The chest x-ray evidence of pulmonary Koch in 100 patients is 60%.The sputum conversion at the end of 1st month is 71%, at the end of 2nd month it is 84% and at the end of 3rd month the same is 92%. In the present study, the infectivity decreases from the baseline with significant P value for sputum conversion of 3+, 2+ and 1+ sputum positivity. Directly Observed Treatment is an effective intervention for improving adherence to tuberculosis treatment programme in a resource-poor country. A significant decrease in conversion rate was observed with the initial high grade smear positive cases.

**More Prospective studies on larger number of patients are necessary to sub-stantiate our findings in this study.**

## INTRODUCTION

Tuberculosis remains a major public health problem worldwide. It has been estimated that someone in the world is newly infected with TB every second, nearly 1% of the world population is infected with TB every year and overall, one third of the world population is infected with Mycobaterium TB^1^,^2^,^3^. In March 1993, the WORLD HEALTH ORGANISATION (WHO) took an unprecedented step & declared TB as a "Global Emergency 4,5. This was the first time, the WHO had ever singled out a disease in this manner, According to an estimate by the WHO, between 1999 and the year 2020 nearly one billion more people will be newly infected, 200 million will get sick and 70 million will die from TB if control measures are not strengthened^4^. The DOTS-Directly Observed Treatment Short Course Chemotherapy strategy for TB control represents one of the major public health strategies of the recent times which have resulted in importance therapeutic breakthrough, not only in our own country, but also all over the world. 8 out of 10 patients under DOTS regimen are cured. Nation wide DOTS covers 632 district and 1114 million people under RNTCP all over the country^6^,^7^,^8^. Pulmonary TB comprises of about 85% of all new TB cases in INDIA and they are responsible for the spread of the infection, therefore Pulmonary TB is epidemiologically important and become the topmost priority from public perspective.

## AIMS AND OBJECTIVES

1. To determine sputum conversion rate at monthly intervals of 1^st^, 2^nd^ and 3^rd^ month of treatment and to compare with second month sputum conversion rate in new smear positive cases (cat-1) under RNTCP.
2. To co-relate x-ray proved TB in smear positive cases.
3. To determine the sputum positivity in different age and sex distribution.

## MATERIAL AND METHODS

The study was conducted at DOTS Center, Gauhati Medical College and Hospital; Guwahati between July 2005 to June 2006.The study is a prospective study and consists of 100 cases of smear positive pulmonary tuberculosis cases (category 1) irrespective of age and sex.

Inclusion criteria:

Newly diagnosed smear positive pulmonary tuberculosis cases (category 1, excluding the seriously ill extra pulmonary tuberculosis cases).

Exclusion criteria:

- Seriously ill extra pulmonary category 1 cases.
- Category II and category III patients.
- Patients lost to follow up.

Drug regimens:

The selected patients were administered antituberculosis drugs under DOTS regimen according to category I i.e. 2H_3_ R_3_ Z_3_ E_3_ & 4H_3_ R_3_.

Three sputum samples are collected over two consecutive days

- Three sputum specimens (spot—morning—spot) are collected over 2 consecutive days
- Spot sample on the first day.
- One early morning sample on second day and
- One spot sample on the second day.

Follow up of the cases and smear examination:

Two sputum specimens (spot—morning) are taken each time for follow-up sputum smear examinations at specified intervals: at the end of the 1st month, at the end of 2nd month and at the end of 3rd month. The intensive phase of treatment consisting of H3R3Z3E3 is continued for another 4 weeks if the patients are positive at the end of 2nd month as per DOTS.

Results and Observations:

The age & sex distribution of 100 patients showed that majority of the patients (74%) belonged to 2^nd^, 3^rd^ and 4^th^ decades with mean age of 34 years and standard deviation of 16 years Table 1 & 75% were males and 25% were female with male to female ratio 3:1 respectively. The chest x-ray of all the 100 Smear Positive patients was done and it was found that only 60 (60%) patients had X-ray evidence suggestive of pulmonary Koch. The correlation between the X-ray and the sputum positivity was observed which shows cavitations, parenchymal and both involvement involving more than one segment in 60% patients with X-ray evidence of pulmonary TB in new sputum smear positive cases in the present study.

**Table I:** Age distribution of patients

Age in years (Range)	No. of patients (%)
11-20	15(15%)
21-30	43(43%)
31-40	16(16%)
41-50	10(10%)
51-60	6(6%)
61-70	8(8%)
71-80	2(2%)
Total	100(100%)

**Table II:** Number of patients of grade 3+,2+,1+ & scanty(SC) sputum positivity at baseline, at the end of 1st, 2nd & 3rd month.

	Baseline	At 1st month	At 2nd month	At 3rd month
3+	42	0	0	0
2+	21	12	7	3
1+	26	14	4	4
SC	11	3	5	1
Neg.*	-	71	84	92

* = negative.

**Fig 1:**
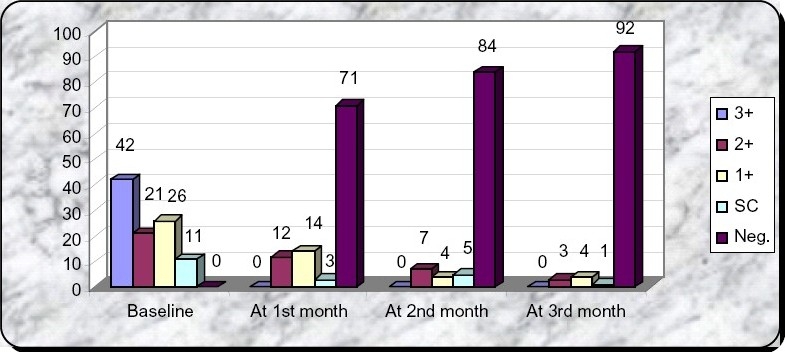
Bar diagram showing sputum positivity at baseline, at the end of 1st, 2nd & 3rd month.

**Table III:** Paired sample test result of sputum conversion of 3+, 2+, 1+ and scanty sputum positivity from baseline and at the end of 1st, 2nd and 3rd month: (Data Shown In Table 2, 3 & 4 & Fig 1).

Sp. Con.*	Baseline	Sp. Con.* at 1st Month	Sp. Con.* at 2nd Month	Sp. Con.* at *3rd Month*
3+ → 2+	42	12(29%)	7(16.5%)	3(7%)
3+ → 1+	42	9(21%)	4(10%)	4(10%)
3+ → SC	42	0	3(7%)	1(12.5%)
3+ → N	42	21(50%)	7(16.5%)	6(14%)
2+ → 1+	21	5(24%)	-	-
2+ → SC	21	0	2(10%)	-
2+ → N	21	16(76%)	3(14%)	2(10%)
1+ → SC	26	3(12%)	-	-
1+ → N	26	23(88%)	3(12%)	-
SC → N	11	11(100%)	-	-
			71(Rem. −ve** at 2nd month)	84(Rem. −ve** at 3rd month)
Total	-	100	100	100

To calculate P value at the end of 1st month for the 3+ sputum positivity 42 patients at baseline are taken as variable 1 and respective sputum conversion form 3+ →2+(12 patients), 3+ → 1+(9 patients), 3+ → scanty (0 patient) and 3+ → N (21 patients) at the end of 1st month are taken as variable 2. Calculated t value and P value for 3+ at the end of 1st month is 7.275 and 0.005 (< 0.05), respectively.

To calculate P value for 3+ sputum positivity at the end of 2nd month, 21 patients whose sputum remains positive of grading 2+, 1+ (12+9 patients respectively) from 3+ at the end of 1st month is taken as variable 1 and sputum conversion from 3+ →2+ (7 patients) 3+ →1+ (4 patients), 3+ → scanty (4 patients) and 3+ → N (7 patients) is taken as variable 2. Calculated t value & P value is 15.280 and 0.001 (< 0.05) respectively.

To calculate P value at the end of 3rd month for 3+ sputum positivity, 14 patients whose sputum remains positive of grading 2+, 1+, Scanty (7+4+3 patients respectively)from 3+ at the end of 2nd month is taken as variable 1 and sputum conversion from 3+ → 2+ (3 patients), 3+ → 1+ (4 patients), 3+ → scanty (1 patients) and 3+ → N (6 patients) is taken as variable 2. Calculated t value and P value is 10.088 and 0.002 (<0.05) respectively.

To calculate P value for 2+ sputum positivity, 21 patients of 2+ sputum positivity at baseline is taken as variable 1 and total patient whose sputum is converted from 2+ → 1+ and 2+ → SC at the end of 1st month (5 patients), at the end of 2nd month (2 patients) and at the end of 3rd month (0 patient) is taken as variable 2. Calculated t value and P value is 12.847 and.006 (<0.05) respectively.

To calculate P value for 1+ sputum positivity, 26 patients at baseline of 1+ sputum positivity is taken as variable 1 and total patient whose sputum is converted from 1+ → scanty and 1+ → Negative at the end of 1st month, at the end of 2nd month is taken as variable 2. Calculated t value and P value is 16.33 and.039 (<.05) respectively.

## SPUTUM CONVERSION

**Fig 2:**
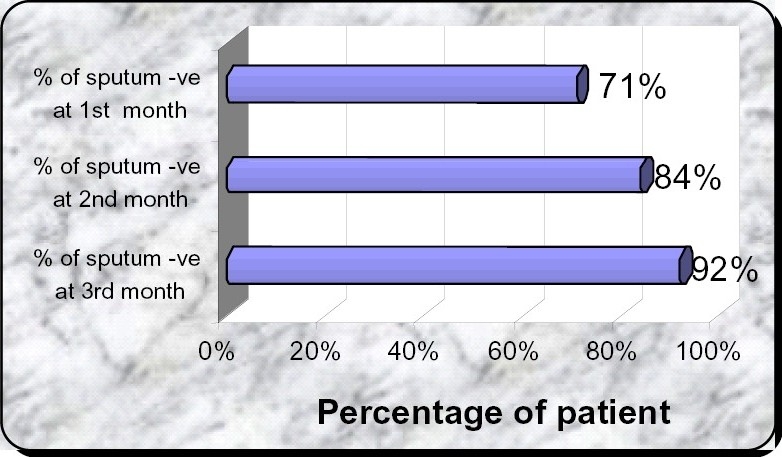
Percentage of the patients whose sputum became negative at the end of 1st, 2nd and 3rd month.

In the present study, sputum conversion i.e. from smear positive to smear negative at the end of the 1st month, 2nd month & 3rd month is 71%,84% and 92% respectively (fig 2).

**Table IV:** Paired samples test result of sputum conversion.

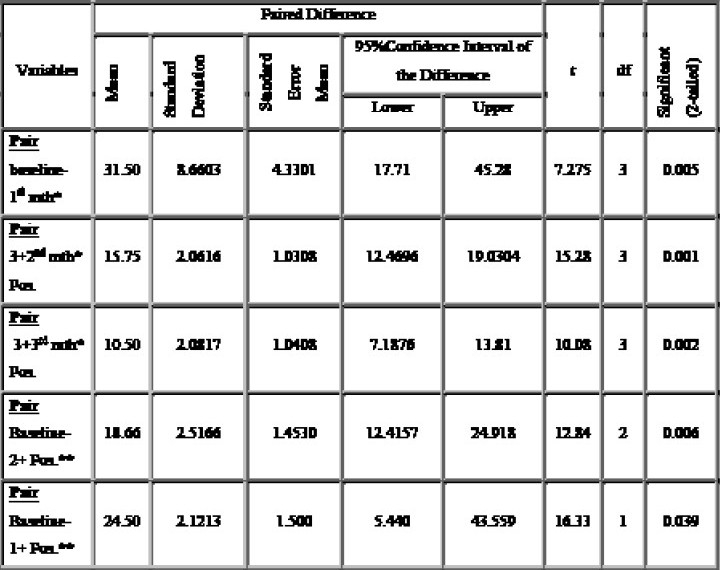

* = month, ** = positivity. (Data calculated using SPSS software)

For calculating the sputum conversion rate^9^,^10^,^11^, the number of smear positive patients who had their sputum converted to smear negative at the end of intensive phase is divided by the number of smear-positive patients started on treatment, and the ratio is multiplied by 100 for obtaining percentage.

Sputum conversion rate=No. of sputum smear-positive converted to sputum smear negative at the end of intensive phase*Total no. sputum smear positive patients initiated on treatment×100

*For calculating sputum conversion rate for new sputum smear positive patients only, all those who converted at the end of IP (at the end of two months) and at the end of extended IP (at the end of three months) should be added to obtain the numerator. This is not applicable for the rest of sputum smear positive patients.

Sputum conversion rate is calculated as per RNTCP guidelines^9^,^10^,^11^ by taking the number of patients who had their sputum converted to smear negative at the end of intensive phase including patients whose sputum was positive at the end of the 2^nd^ month but negative at the end of 3^rd^ month divided by the number of smear positive patients stated on treatment. The ratio is multiplied by 100 for obtaining percentage.

In the present study of 100 smear positive pulmonary tuberculosis cases, 84(84%) patients at the end of 2^nd^ month and 92 (92%) patient at the end of 3^rd^ month were smear negative.

Sputum conversion Rate=92100×100=92%.

## DISCUSSION AGE & SEX DISTRIBUTION

The age & sex distribution of 100 patients showed that majority of the patients (74%) belonged to 2^nd^, 3^rd^ and 4^th^ decades with mean age of 34 years and standard deviation of 16 years & 75% were males and 25% were female with male to female ratio 3:1 respectively. As per WHO Report 2006(country file INDIA), Age and sex distribution provided for a subset of new smear-positive cases notified in 2004 showed that maximum cases reported varies from 1^st^ decade to 4^th^ decade and with male predominately affected^7^. The present study also shows the same result.

## CHEST X-RAY EVIDENCE

The chest x-ray of all the 100 Smear Positive patients was done and it was found that only 60 (60%) patients had X-ray evidence suggestive of pulmonary Koch. The correlation between the X-ray and the sputum positivity was observed which shows cavitations, parenchymal and both involvement involving more than one segment in 60% patients with X-ray evidence of pulmonary TB in new smear positive cases. According to Toman et al, microscopy (98%) is a more specific test than X-ray (50%) for TB diagnosis and Microscopy (98%) is more objective and reliable than X-ray (70%).There is considerable overlaps between primary and post-primary TB on a chest X-ray. But the following points favor post-primary TB: predilection for upper lobe involvement, propensity for cavitations & rarity of lymphadenopathy^12^. According to Fraser et al^13^, a typical fibroproductive lesion may look inactive but show active granulomatous inflammation and contain viable bacilli. According to Woodring et al^14^, more than one segment is involved, cavitations occurs in 40%-87% cases and mixed exudative and fibroproductive lesion is the commonest finding (79%). Endobronchial spread is seen in a CXR in 19% to 58% cases^15^ & by HRCT in up to 98% cases^16^.

## SPUTUM CONVERSION

**Fig 3:**
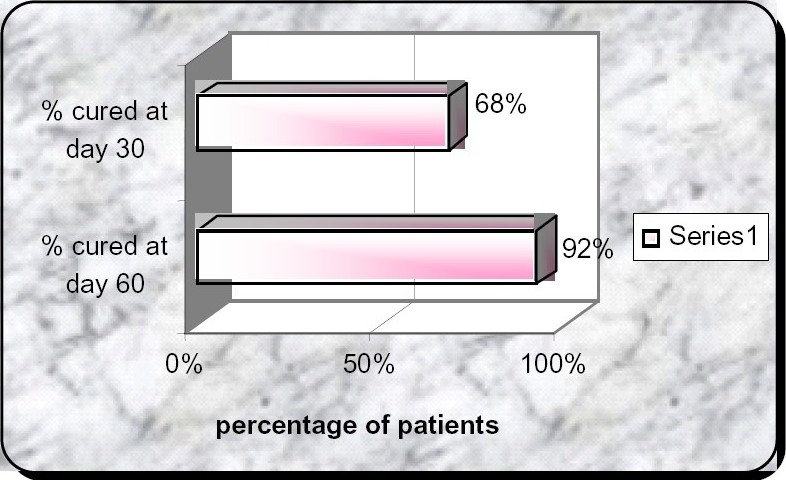
Percentage responders at the end of the study (Baruwa P et al)

The best way to monitor the treatment results of a pulmonary smear positive case is to check for the conversion of sputum from smear positive to smear-negative^17^–^25^. In the present study, among 100 cases of sputum positive pulmonary tuberculosis (category-I) patients,71(71%) patients become smear negative at the end of the 1^st^ first month, 84 (84%) patients become sputum smear negative at the end of 2^nd^ month and 92 (92%) patient become sputum smear negative at the end of 3^rd^ month. The present study match with the study of Baruwa et al^26^ (April 2005) which shows sputum conversion of 68% at the end of 1^st^ month. The study done by Baruwa et al^26^ was also same four drugs in fixed dose combination in sputum Positive Indian Patients and included 175 patients.(Fig.3).

The present study shows infectivity decreases from baseline at the end of 1^st^ month, 2^nd^ month and 3^rd^ month. At the end of 1st month, 42 patients whose sputum was positive of 3+ grading for AFB microscopy examination, 21 (50%) patients become sputum smear negative, 12(29%) patient's sputum converted from 3+ to 2+ grading and 9 (21%) patients sputum converted from 3+ to 1+ grading. P value calculated using the variables is.005 (<.05, significant) with 95% confidence interval of 17.71 to 4.5.28 and standard error mean of 4.3301. At the end of 2nd month, among remaining 21 patients whose sputum was positive at the end of 1st month from 3+, 7(16.5%) patients remained 2+ sputum positive, 4(10%) patient's sputum converted from 3+ to 1+ grading, 3 (7%) patient's sputum converted from 3+ to scanty and 7(16.5%) patients were sputum smear negative. P value calculated using the variables is.001 (<.05, significant) with the 95% confidence interval of 12.46 to 19.03 and standard error mean of 1.03. At the end of 3rd month, 14 patients whose sputum was positive at the end of 2nd month from 3+, 3(7%) patient's sputum remained 2+, 4 (10%) patient's sputum converted from 3+ to 1+, 1 (2.5%) patient's sputum converted from 3+ to scanty and 6(14%) patient were sputum smear negative. Calculated P value using the variables is.002 (<0.05, significant) with 95% confidence interval of 7.18 to 6 to 13.81 and standard error mean of 1.04.

For 21 patients, where sputum smear positivity was 2+ at the baseline, sputum conversion of 5(24%) patient converted from 2+ to 1+ and 16 (76%) patients were smear negative at the end of 1st month. Among 5 patients, 2(10%) patient's sputum converted from 2+ to scanty and 3(14%) patients were sputum smear negative at the end of 2nd month. The remaining 2(10%) patient's sputum smear turned negative at the end of 3rd month. With 21 patients at baseline and patient's sputum conversion from 2+ at 1st month, 2nd month and 3rd month, calculated P values is.006 (<0.05, significant), 95% confidence interval of 12.41 to 24.91 and standard error mean of 1.45.

Among 26(26%) patient of 1+ sputum smear positivity at baseline, 3(12%) patients were sputum converted from 2+ to scanty and 23(88%) patients were sputum smear negative at the end of 1st month. The remaining 3(12%) patient's sputum smear turned negative at the end of 2nd month. Using the variables, P values is.039 (<0.05, significant) with 95% confidence interval of 5.440 to 43.559 and standard error mean of 1.50.

All 11(100%) patient of scanty sputum smear positivity at baseline turned negative at the end of 1st month.

Rutta et al^27^ from Tanzania (July 2001) showed sputum conversion after the 2 month intensive phase was 88%.

Rieder et al^28^ from Paris (April 1996) showed sputum conversion of 75.0% with a range from 61.7% to 90.9% in patients with initially strongly and weakly positive smear respectively after the 2 month intensive phase. In the study, it was concluded sputum smear results at two months strongly predict bacteriologic results beyond three months of treatment, and thus identify cases that might benefit from a prolongation of the intensive phase.

Lienhardt et al^29^ from Gambia (September, 1998) observed sputum smear conversion at the end of 2 months after the start of treatment in 90% of smear positive cases and was more likely occurs if the initial bacterial load in the sputum was low.

The present study is comparable with study of Rutta E et al 27 (July 2001), Rieder HL et al 28 (April 1996) and Lienhardt C et al 29 (September, 1998).

In the present study, 8(19.5%) patients whose sputum smear examination was positive of 3+ grading at baseline remained positive at the end of 3rd month of grading 2+ [3(7%) patients],1+ [4(10%) patients] and scanty [1(2.5%) patients].

Rajpal et al^30^ from New Delhi (2002) reveals that patients with 3+ sputum smear grading not only require extension of treatment in the intensive phase more often than those with scanty, 1+ or 2+ grading but also have significantly higher failure rate.

Singla et al^31^ from New Delhi in (2003) concluded that age group 41-60 years and more than 60 years. Presence of numerous bacilli on initial sputum smear examination, and multiple cavitary disease were the significant factors associated with persistent sputum positivity at the end of 2 months of treatment (P< 0.0001).

Zhao et al^32^ from China (1997) reveals 95% patients' sputum converted in the third month of treatment. They concluded sputum conversion during the third month of treatment is an important predictor of treatment success, failure to convert predicts treatment failure.

Singla et al^33^ from New Delhi (2005) in the study, highest grading of sputum smear examination was recorded. Sputum conversion rates among patients graded as 3+ and rest of the patients (combined graded sputum 1+ and 2+) at the end of two months were 62.2% and 76.8% respectively (p<0.0001), and at the end of three months were 81.3% and 89.5% respectively (P<0.0001). They concluded that smear positive patients with heavy bacillary load showed statistically significant poor sputum conversion rates at two and three months and higher failure rates as compared to patient with lesser bacillary load.

The present study is comparable with study of Rajpal et al^30^ (2002), Singla et al^31^ (2003) and Singla et al^33^ (2005).

### Sputum conversion rate

In the present study, sputum conversion at the end of 1st month is 71%, at the end of 2nd month in 84% and at the end of 3rd month is 92%. Sputum conversion rate of the present study calculated is 92%.

### Summary & conclusion

- In conclusion, the overall sputum conversion rate under Directly Observed Treatment, Short Course (DOTS) chemotherapy in 100 sputum smear positive Pulmonary Tuberculosis in DOTS centre, Gauhati Medical College & Hospital was 92%.
- The chest x-ray evidence of pulmonary Koch in present study in 100 category-1 new smear positive pulmonary TB patients is 60%.
- In the present study, age distribution of 100 patients showed that majority of the 74 (74%) patients belonged to 2nd, 3rd and 4th decades with mean age of 34 years and standard deviation of 16 years. Sex distribution results showed 75 (75%) were male and 25(25%) were female with a sex ratio of 3:1.
- The sputum conversion at the end of 1st month is 71%, at the end of 2nd month it is 84% and at the end of 3rd month the same is 92%.
- In the present study, the infectivity decreases from the baseline with significant P value for sputum conversion of 3+, 2+ and 1+ sputum positivity. Directly Observed Treatment is an effective intervention for improving adherence to tuberculosis treatment programme in a resource-poor country.
- A significant decrease in conversion rate was observed with the initial high grade smear positive cases.

More Prospective studies on larger number of patients are necessary to substantiate our findings in this study.

(Received thesis grant from RNTCP. Authors thankful to RNTCP for providing thesis grant)
